# Next-generation sequencing survey of acute febrile illness in Senegal (2020–2022)

**DOI:** 10.3389/fmicb.2024.1362714

**Published:** 2024-04-09

**Authors:** Gregory S. Orf, Ambroise D. Ahouidi, Maximillian Mata, Cyrille Diedhiou, Aminata Mboup, Abdou Padane, Noel Magloire Manga, Agbogbenkou Tevi Dela-del Lawson, Francisco Averhoff, Michael G. Berg, Gavin A. Cloherty, Souleymane Mboup

**Affiliations:** ^1^Core Diagnostics, Abbott Laboratories, Abbott Park, IL, United States; ^2^Abbott Pandemic Defense Coalition, Abbott Park, IL, United States; ^3^Institut de Recherche en Santé, de Surveillance Epidémiologique et de Formation, Dakar, Senegal; ^4^Unit of Infectious and Tropical Diseases, Université Assane Seck, Hôpital de la Paix, Ziguinchor, Senegal; ^5^Unit of Infectious and Tropical Diseases, Hôpital Mame Abdou Aziz Sy Dabakh, Tivaouane, Senegal

**Keywords:** Senegal, acute febrile illness, next-generation sequencing, viral enrichment, malaria

## Abstract

**Introduction:**

Acute febrile illnesses (AFI) in developing tropical and sub-tropical nations are challenging to diagnose due to the numerous causes and non-specific symptoms. The proliferation of rapid diagnostic testing and successful control campaigns against malaria have revealed that non-*Plasmodium* pathogens still contribute significantly to AFI burden. Thus, a more complete understanding of local trends and potential causes is important for selecting the correct treatment course, which in turn will reduce morbidity and mortality. Next-generation sequencing (NGS) in a laboratory setting can be used to identify known and novel pathogens in individuals with AFI.

**Methods:**

In this study, plasma was collected from 228 febrile patients tested negative for malaria at clinics across Senegal from 2020–2022. Total nucleic acids were extracted and converted to metagenomic NGS libraries. To identify viral pathogens, especially those present at low concentration, an aliquot of each library was processed with a viral enrichment panel and sequenced. Corresponding metagenomic libraries were also sequenced to identify non-viral pathogens.

**Results and Discussion:**

Sequencing reads for pathogens with a possible link to febrile illness were identified in 51/228 specimens, including (but not limited to): *Borrelia crocidurae* (N = 7), West Nile virus (N = 3), *Rickettsia felis* (N = 2), *Bartonella quintana* (N = 1), human herpesvirus 8 (N = 1), and Saffold virus (N = 1). Reads corresponding to *Plasmodium falciparum* were detected in 19 specimens, though their presence in the cohort was likely due to user error of rapid diagnostic testing or incorrect specimen segregation at the clinics. Mosquito-borne pathogens were typically detected just after the conclusion of the rainy season, while tick-borne pathogens were mostly detected before the rainy season. The three West Nile virus strains were phylogenetically characterized and shown to be related to both European and North American clades. Surveys such as this will increase the understanding of the potential causes of non-malarial AFI, which may help inform diagnostic and treatment options for clinicians who provide care to patients in Senegal.

## Introduction

The myriad causes of acute febrile illness (AFI) in West Africa have been difficult to quantify (Maze et al., 2018), in part due to the lack of a formal case definition (Crump, 2014; Rhee et al., 2019). While malaria (*Plasmodium* spp.) traditionally has been the most common suspect, its incidence has been steadily decreasing due to aggressive control campaigns and wide distribution of effective treatments (D’Acremont et al., 2010; Murray et al., 2012; World Health Organization [WHO], 2022). The introduction of malaria rapid diagnostic tests (RDTs) has additionally indicated that a considerable proportion of AFI cases are non-malarial in nature (Reyburn et al., 2004; Thiam et al., 2011; Wainaina et al., 2022). The prevalence and severity of AFI are influenced by population structure and common comorbidities (Maze et al., 2018), but also local climate and ecology, which dictate the spatiotemporal distribution of vector and reservoir species for a variety of common zoonotic pathogens (Campbell et al., 2015; Ryan et al., 2015; Carlson et al., 2016, 2023).

West Africa is one of the most climatologically diverse regions on the planet, ranging from the arid Sahara Desert to tropical moist deciduous forests in a north-south span of only 1,000 km (Nicholson, 2018). The southern part of the region is strongly influenced by a June-to-October monsoon and is prone to natural hazards such as floods, droughts, and heat waves (Nicholson, 2013; Raj et al., 2019). Numerous surveys have linked these climatic boundaries to the natural ranges of pathogen-carrying ticks, mosquitoes, and other hematophagous insects; for example, *Ornithidoros* ticks carrying *Borrelia* spp. prefer latitudes receiving less than 750 mm of rainfall per year (Trape et al., 1996, 2013) and *Plasmodium* spp. require sustained temperatures under 25–29°C to optimally transmit (Mordecai et al., 2013; Villena et al., 2022).

Since targeted diagnostics for non-malarial AFIs are rarely available in clinics, next-generation sequencing (NGS) in a research laboratory setting can be used to identify known and novel pathogens in individuals with AFI. A major benefit of this technology is its ability to multiplex large cohorts of specimens (Radford et al., 2012; Chiu, 2013; Kapuscinski et al., 2021; Ko et al., 2022). This can be performed in an unbiased fashion (metagenomics; mNGS) to surveille or discover any pathogen present in a specimen (Chiu and Miller, 2019; Ko et al., 2022), or it can be targeted via targeted enrichment (teNGS) to expedite identification of known pathogens, even at low concentration (O’Flaherty et al., 2018; Yamaguchi et al., 2018; Deng et al., 2020; Orf et al., 2021).

The Republic of Senegal reports well over one million cases of AFI per year (Thiam et al., 2011). It straddles three major climatic zones of West Africa (Sahel tropical shrubland, tropical dry forest, and tropical moist deciduous forest) which risk boundary changes, and thus range changes for disease-vectoring arthropods (Carlson et al., 2023), due to climate warming trends and desertification (Evans and Munslow, 2021). Since 2007, malaria RDTs have supplemented clinical assessment in screening AFI patients, leading to a case management algorithm that recommends broad-spectrum antibiotics and antipyretics for patients testing negative for malaria (Thiam et al., 2011). However, in the face of biological and sociological factors such as antimicrobial resistance and urbanization, the etiologies of AFI in Senegal should be documented as broadly as possible to keep clinicians and public health authorities informed and motivate updated case management algorithms. The timely reporting of the circulation of any new or re-emerging pathogens will also be especially important. In this study, we performed both teNGS and mNGS to identify viral and non-viral pathogens, respectively, in a cohort of prospectively collected plasma specimens from 228 Senegalese AFI patients.

## Materials and methods

### Patients and specimen collection

Plasma specimens were collected as part of a prospective pathogen discovery and surveillance study. Three clinics located in Bounkiling, Ziguinchor, and Tivaouane were utilized as collection sites. The investigation protocol was approved by the National Ethics Committee for Health Research (Comité National d’Ethique pour la Recherche en Santé; CNERS) of the Senegalese Ministry of Health, under approval number 000129/MSAS/CNERS.

During the enrollment periods, individuals suspected of an acute febrile illness (including recent or current fever ≥ 38 C, without significant respiratory symptoms) were screened in the clinic by malaria RDTs (SD Bioline Malaria Ag Pfal, detecting HRP2 protein) provided by the Ministry of Health, and any individuals testing negative were offered enrollment in the study. An accounting of the testing numbers and an evaluation of the sample size can be found in the Supplementary Information. Additionally, the Tivaouane site was a regional hospital with available capacity to perform COVID-19 molecular diagnostics in a core laboratory; individuals screened at this site were only considered for enrollment after receiving a negative COVID-19 test. Written informed consent for participation was provided by the enrollees, or by their legal guardians/next of kin. Physical data (*i.e.*, age, sex, and weight) and symptomatology were collected alongside a venous blood draw between 2.0–3.5 ml stored in EDTA tubes. Plasma was separated by centrifugation. All enrollee metadata and plasma were de-identified prior to laboratory analysis.

### Nucleic acid extraction and preparation of NGS libraries

Aliquots of each plasma specimen were pre-treated with Benzonase nuclease (> 250 units/μl, Sigma-Aldrich, St. Louis, MO) at 37 C for 3 h to decrease the abundance of human background nucleic acids (718 μl plasma + 80 μl of 10 × buffer + 2 μl Benzonase). Total nucleic acids (TNA) were extracted from 500 μl of each Benzonase-treated plasma specimen on an *m*2000sp system using a laboratory-defined assay (Abbott Molecular, Des Plaines, IL). The automated extraction protocol uses magnetic microparticles for capture and modified *m*2000 buffers to recover both RNA and DNA (Orf et al., 2021). Each extraction contained four positive controls (viral stocks spiked into HIV-positive plasma at 3.0 log copies/ml), one negative control (normal human plasma), and one no-template control (phosphate-buffered saline) per ninety primary specimens.

The RNA component of the resulting TNA was reverse-transcribed using SuperScript IV reverse transcriptase for first-strand synthesis and Sequenase v2 polymerase for second-strand synthesis (ThermoFisher Scientific, Waltham, MA). The resulting DNA/cDNA was purified using AMPure XP cleanup beads (Beckman Coulter, Indianapolis, IN) and converted into NGS libraries using a Nextera XT kit in conjunction with *IDT for Illumina* non-biotinylated unique dual index barcode adapters (Illumina, San Diego, CA). The resulting libraries were assessed for size and concentration using a TapeStation 4200 system (Agilent Technologies, Santa Clara, CA) and Qubit Flex fluorimeter (ThermoFisher Scientific, Waltham, MA, USA), respectively.

### Viral target enrichment

An aliquot of each metagenomic library was subjected to viral sequence enrichment using the Comprehensive Viral Research Panel (CVRP; Twist Biosciences, South San Francisco, CA, USA) (Berg et al., 2020). Briefly, libraries were pooled on an equimolar basis in sets of 16–24 (each set containing a positive and negative control) such that each pool contained ∼3 μg total DNA, as assessed by a Qubit Flex fluorimeter. The pools were dried down using a vacuum centrifuge, then resuspended in a solution of Human Cot-1 DNA and xGen Universal Nextera Blockers (Integrated DNA Technologies, Coralville, IA). Hybridization of the CVRP probes to the NGS libraries was performed per manufacturer’s instructions for 16 hr. Hybridized sequences were separated from non-hybridized sequences by affinity interaction on Streptavidin beads (Twist Biosciences), amplified using a KAPA library amplification kit (Roche, Basal, Switzerland), and re-purified using magnetic PCR beads (Twist Biosciences). The resulting libraries were analyzed for size and concentration as above.

### NGS and bioinformatic analysis

Groups of 48 CVRP-enriched libraries (*e.g.*, two enrichment pools) were multiplexed for sequencing (target-enriched sequencing; teNGS) on a MiSeq instrument using a MiSeq v2 300 cycle kit (Illumina, San Diego, CA), while groups of 48 unbiased metagenomic libraries were multiplexed for sequencing (metagenomics; mNGS) on a NextSeq 1000 instrument using a P2 300 cycle kit (Illumina, San Diego, CA). The resulting FASTQ files were uploaded to either the SURPI pipeline (Naccache et al., 2014) or in-house “DiVir3” pipeline for bioinformatic analysis, including known and divergent pathogen read identification. An in-house implementation of BLAST (Altschul et al., 1990) was also utilized for sequence comparison with nucleotide and protein databases. Specific pathogen reads in each specimen were normalized per million total sequenced reads (“reads per million”; RPM) to compare across different specimens. RPM for each pathogen in each specimen was compared against the RPM of the same pathogen in any no-template control; this value, called “RPM ratio” (*RPM-r*) or “RPM fold-above controls” here, was used to gauge the probability of the pathogen being truly present (Miller et al., 2019). Any specimen with a pathogen showing *RPM-r* > 10 was further investigated via read mapping in CLC Genomics Workbench (Qiagen Corp., Germantown, MD, USA) to confirm that at least three regions of the pathogen’s genome was covered with high-quality paired-end reads.

### Phylogenetic tree reconstruction

For phylogenetic analysis of viral genomes of interest, sets of closely-related genomic sequences were retrieved from GenBank and aligned using the L-INS-i algorithm implemented in MAFFT version 7.487 (Katoh and Standley, 2013). Phylogenetic reconstruction using the maximum likelihood (ML) method was performed using IQTREE version 2.1.3 (Minh et al., 2020). Briefly, the ModelFinder algorithm (Kalyaanamoorthy et al., 2017) was first used to automatically find a suitable nucleotide substitution model using Bayesian Information Criterion as a scoring method. Initial ML tree reconstruction was achieved using a stochastic algorithm and then optimized using the Nearest Neighbor Interchange (Robinson, 1971; Moore et al., 1973) heuristic method. The tree with the best log-likelihood score was retained, and branch supports were provided using 1,000 replicates of Ultrafast Bootstrapping (Hoang et al., 2018). Inference of rooted molecular clock phylogenies and elimination of temporal outliers were iteratively performed using TreeTime v.0.10.1 (Sagulenko et al., 2018). After all temporal outliers were identified and removed, a discrete state phylogeographic analysis was also performed using TreeTime. Output trees were visualized and annotated with associated metadata using the *ggplot2* and *ggtree* packages (Wickham, 2016; Yu et al., 2017) for the *R* programming language.

### Use of public domain data

Global administration zone shapefiles were obtained from Natural Earth via www.naturalearthdata.com (accessed on 2023-05-25). Global ecological zone shapefiles were obtained from the map catalog of the Food and Agriculture Organization of the United Nations via data.apps.fao.org/map/catalog/ (accessed on 2023-05-25). Historic meteorological data (particularly daily air temperature and precipitation data from Ziguinchor and Diourbel weather stations) was obtained from the National Centers for Environment Information of the United States National Oceanic and Atmospheric Administration via^1^ (accessed on 2023-06-29). These datasets are public domain and may be reproduced without permission. All data utilized in this study, unless otherwise denoted, were organized and visualized using the *tidyverse* and *ggplot2* packages for the *R* programming language (Wickham, 2016).

## Results

### Febrile patients were enrolled across three sites over 3 years

**FIGURE 1:**
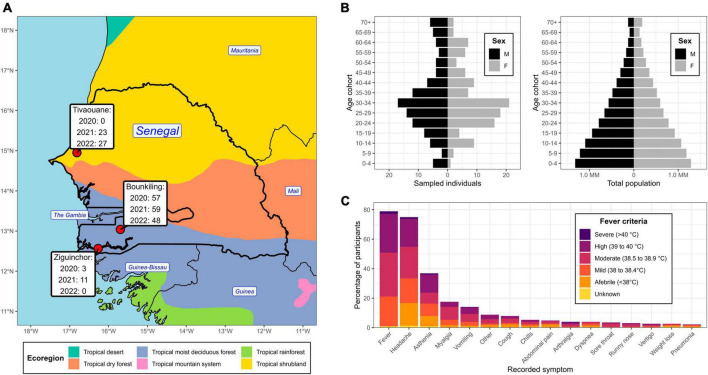
Geographic location, demographics, and symptom profile of the study population. **(A)** Partial map of western Africa, centered on Senegal, overlaid with the year 2010 ecological zones (ecoregions) as defined by the United Nations. The clinic locations are highlighted, with a temporal breakdown of enrollee count. **(B)** Demographic pyramid of the enrollees (left) and total national population (right). **(C)** An accounting of the recorded symptoms from enrollees and their relation to fever criteria at the time of clinical intake.

From 2020 to 2022, individuals complaining of symptoms consistent with acute febrile illness were received at clinics at three locations in Senegal: Bounkiling, Ziguinchor, and Tivaouane (Supplementary Table 1). The clinics span two major ecological regions (Figure 1A): Tivaouane is situated in the Sahel tropical shrubland about 100 km to the northeast of the capital Dakar, and Bounkiling and Ziguinchor in the southwestern tropical deciduous forest. Study enrollment occurred from October 2020-July 2022 in Bounkiling, from November 2020-April 2021 in Ziguinchor, and from August 2021-July 2022 in Tivaouane. Patients were administered malaria RDTs (during the enrollment period−Bounkiling: 3,597; Ziguinchor: 1,003; Tivaouane: 2,537) and those determined to be negative by a clinician (cumulatively 5,925/7,137; 83%) were considered for enrollment in this study. Overall, the malaria positivity rate in the clinics during the enrollment periods was much higher in the southwestern deciduous forest region (Bounkiling/Ziguinchor; 25.3% positivity) than in the northern shrubland region (Tivaouane; 1.8% positivity). Across the three sites, 228 individuals in total agreed to be enrolled (Bounkiling: 164; Ziguinchor: 14; Tivaouane: 50). Additionally, all patients enrolled from Tivaouane received negative COVID-19 diagnostic test results at the clinic.

There was an even split between males and females amongst the cohort (50.4% and 49.6%, respectively), with average and median ages of 33.7 yr and 30.0 yr, respectively (Figure 1B). There was an underrepresentation of younger age groups (< 20 yr) compared to the national demographic structure (Figure 1B), however, the clinicians at the sites intentionally focused their collections on adults. At time of blood draw, 47 enrollees presented with a body temperature below 38°C (despite recent history of fever ≥ 38°C), with the most noted symptoms being headache and asthenia (Figure 1C). One-hundred fourteen enrollees (50%) presented with a body temperature between 38.0–38.9 C (*i.e.*, mild to moderate fever) and 64 enrollees (28%) presented with a body temperature over 39 C (*i.e.*, high to severe fever). Overall, the most noted symptoms across the cohort were headache, fatigue, body aches, and vomiting. Respiratory symptoms (*e.g.*, cough) were observed in 24 enrollees and 4 enrollees showed severe neurological signs or symptoms (*i.e.*, coma).

### Eight species of virus, four of bacteria, one of fungus, and two of parasite were detected by NGS

Using teNGS and mNGS approaches in tandem, we recovered sequencing reads corresponding to a known cause of AFI in 51/228 (22.4%) of specimens, of which four cases (4/50; 8.0%) were dual infections (Table 1 and Supplementary Figures 1–3). In Bounkiling, 39 cases (39/164; 23.8%) had one or more pathogens identified, in Ziguinchor, 4 cases (4/14; 28.6%) had a pathogen identified in NGS, and only 8 cases (8/50; 16.0%) had a pathogen identified in Tivaouane.

**TABLE 1:** Pathogens detected by NGS and the recorded symptoms in the affected patients.

Pathogen type	Pathogen	Specimens	Recorded sign or symptom
Viral	West Nile virus	BK045, **BK160**, FZ008	Fever > 38 C (2/3; 66.7%) Headache (2/3; 66.7%) Fatigue (1/3; 33.3%) Shortness of breath (1/3; 33.3%)
	Rotavirus A	BK080, BK183	Headache (2/2; 100%) Fever > 38 C (1/2; 50%) Fatigue (1/2; 50%) Muscle aches (1/2; 50%) Vomiting (1/2; 50%)
	HIV-1	**BK046**, **BK262**, BK387, FZ007, TV022	Fever > 38 C (2/5; 40%) Headache (2/5; 40%) Cough (2/5%; 40%) Neurological (vertigo or convulsion; 2/5; 40%)
	Hepatitis B virus	BK040, BK120, BK245, **BK262**, BK263, BK307, BK315, **TV012**, TV023, TV039	Fever > 38 C (9/10; 90%) Headache (6/10; 60%) Fatigue (4/10; 40%) Muscle aches (2/10; 20%) Vomiting (2/10; 20%)
	Hepatitis C virus	BK281	Fever > 38 C Headache Muscle aches
	Human gammaherpesvirus 8	FZ006	Fever > 38 C
	Saffold virus	BK399	Fever > 38 C Cough Nasal discharge
	SARS-CoV-2	BK247	Fever > 38 C Headache Muscle aches
Bacterial	*Acinetobacter baumanii*	**BK099, BK160**	Fever > 38 C (2/2; 100%) Headache (2/2; 100%) Fatigue (1/2; 50%)
	*Bartonella quintana*	BK049	Fever > 38 C Vomiting
	*Borrelia crocidurae*	BK020, BK106, BK232, **BK324**, TV032, TV045, TV050	Fever > 38 C (6/7; 85.7%) Headache (6/7; 71.4%) Muscle aches (3/7; 42.9%) Vomiting (2/7; 26.8%) Fatigue (1/7; 14.3%)
	*Rickettsia felis*	**BK208**, **TV012**	Fever > 38 C (1/2; 50%) Headache (1/2; 50%) Fatigue (1/2; 50%) Shortness of breath (1/2; 50%)
Fungal	*Aureobasidium pullulans*	**BK046, BK099**	Fever > 38 C (1/2; 50%) Headache (1/2; 50%) Fatigue (1/2; 50%)
Parasitic	*Loa loa*	**BK324**	Headache Vomiting
	*Plasmodium falciparum*	BK090, BK091, BK092, BK093, BK094, BK096, BK097, BK207, **BK208**, BK209, BK234, BK285, BK286, BK295, BK386, BK388, BK389, FZ001, TV008	Fever > 38 C (14/19; 73.7%) Headache (13/19; 68.4%) Fatigue (8/19; 42.1%) Muscle aches (2/19; 10.5%) Vomiting (2/19; 10.5%)

Specimen designations have alphabetic prefixes denoting the site that they were collected from: BK, Bounkiling; FZ, Ziguinchor; TV, Tivaouane. Those in bold were found to be dually infected.

**FIGURE 2:**
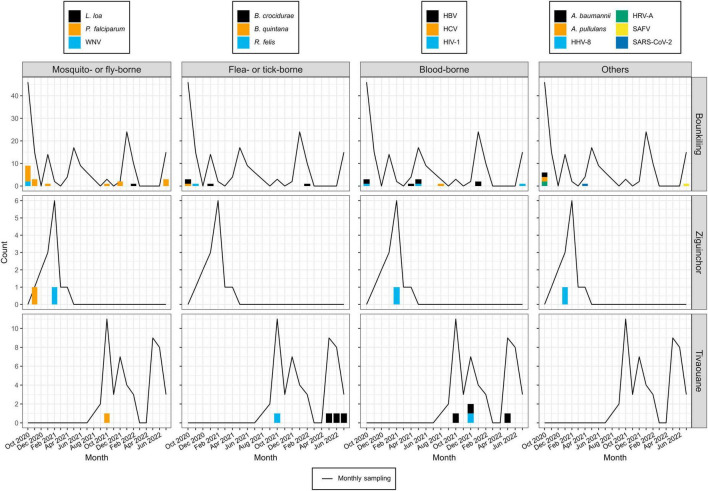
Pathogen detections during the study period, binned by month, site, and pathogen type. Pathogens detection counts are shown as bar plots. Each color legend is specific to its column (*i.e.*, pathogen type). The numbers of specimens collected each month at each site are shown as a line plot (*i.e.*, in months where this value is zero, this means no patients were enrolled, not necessarily that no patients presented with fever; see Supplementary Table 1 for full details).

Despite pre-screening by malaria RDTs, the most common pathogen found across the cohort was *Plasmodium falciparum* (19/50 cases; 38.5%), with most occurring at the southern Bounkiling (17/19; 89.4%) site (Figure 2). Leftover plasma aliquots of these specimens were retested with the same model of RDTs in the laboratory, all returning positive, indicating user error or incorrect specimen segregation at the clinical site. These 19 specimens were nonetheless retained in the cohort to assess potential co-infections. Amongst these malaria cases, 14/19 (73.7%) had a fever above 38°C (14/19; 73.7%) at the clinic (though all complained of a recent history of fever), 13/19 (68.4%) complained of headache, and 8/19 (42.1%) complained of fatigue.

One other mosquito-borne pathogen was detected: West Nile virus (WNV), twice at Bounkiling and once at Ziguinchor. Other pathogens transmitted by non-mosquito arthropods were also identified. Of these, *Borrelia crocidurae*, the cause of Tick-Borne Relapsing Fever (TBRF) in West Africa was definitively detected three times in Bounkiling and three times in Tivaouane. A seventh low-confidence hit to the *Borrelia* genus (28 reads) was also identified in a Bounkiling patient (BK020), with species such as *B. crocidurae*, *B. duttoni*, and *B. recurrentis* represented among classified reads; however, the established geographic ranges of these species (Trape et al., 2013) suggest that this is most likely a *B. crocidurae* infection. Amongst the seven patients with *B. crocidurae* infection, the most reported symptoms were mild fever, headache, and fatigue. The louse-vectored *Rickettsia felis* (flea-borne spotted fever) and *Bartonella quintana* (trench fever) were also detected: *R. felis* once in Bounkiling and once in Tivaouane, and *B. quintana* once in Bounkiling.

Though likely unrelated to AFI, the chronic bloodborne infection Hepatitis B virus (HBV) was detected 10 times (7 times in Bounkiling and 3 times in Tivaouane), human immunodeficiency virus 1 (HIV-1) was detected 5 times (3 times in Bounkiling, and one time in both Ziguinchor and Tivaouane), and Hepatitis C virus (HCV) was detected once in Bounkiling (Figure 2). Patients were not denied enrollment into this study based on HIV/HBV/HCV status.

We detected human herpesvirus 8 (HHV-8; also known as Kaposi’s sarcoma-associated herpesvirus) in one specimen from Ziguinchor (FZ006). Between viral target enrichment and metagenomic NGS, over 40,000 reads were recovered for HHV-8 in this specimen, allowing for > 97% coverage of its ∼140 kb genome. HIV-1 was not detected in this individual by NGS. HHV-8 is endemic in sub-Saharan Africa and may cause fever upon primary infection or be latently reactivated (possibly resulting in sarcoma) in certain scenarios (Andreoni, 2002). Its genome was found to be 99% identical at the nucleotide level to strains recently recovered from Cameroonians (Marshall et al., 2022). Saffold virus (SAFV), recently discovered in 2007, was also detected in a single specimen from Bounkiling from a patient with fever and respiratory symptoms such as cough. This virus has been observed in association with fever, respiratory tract infections, or diarrhea, though its overall prevalence and relationship with humans is still not well-established. This specimen of SAFV is most closely related (88% nucleotide identity) to strains of a novel subtype recently detected in the gut virome of diarrheic Cameroonian patients (Yinda et al., 2019).

Though respiratory, enteric, and opportunistic pathogens are not optimally detected in plasma, SARS-CoV-2 was found once in Bounkiling from a patient (BK247) received in April 2021; despite only recovering 8% of the genome, enough coverage in the spike protein was present to putatively assign it to the Alpha variant (lineage B.1.1.7), which was indeed present in the country at the time (Ahouidi et al., 2021; Perez et al., 2022). We also identified human rotavirus A (HRV-A) infections in two adults from Bounkiling (BK080 and BK183); of these two, the patient corresponding to specimen BK183 presented to the clinic with fever and vomiting, consistent with rotavirus infection. The genomes of neither strain could be fully assembled, though they each bear the highest homology to HRV-A genotype G1P[8] strains from Europe, North Africa, and the United States. Lastly, two opportunistic pathogens were tentatively assigned in three specimens from Bounkiling, all in cases of coinfections. BK046 and BK099 returned reads for the fungus *Aureobasidium pullulans* (BK046 was co-infected with HIV-1) and specimens BK099 and BK160 returned reads for the bacterium *Acinetobacter baumannii*. While reads corresponding to these pathogens were both found at low levels throughout the cohort (Supplementary Figures 1–3), when compared to the background level, these three specimens returned well over 100-fold higher specific mNGS reads per million sequenced than any negative control.

### Mosquito-vectored pathogens were typically detected just after the conclusion of the rainy season

**FIGURE 3:**
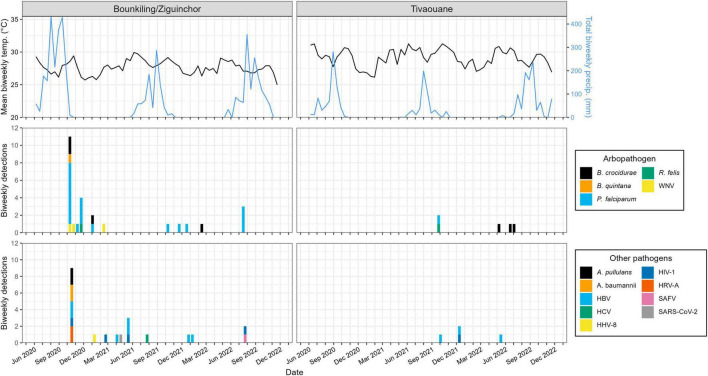
Relationship between pathogen detections and historical meteorological data. The detections outlined in Figure 2 are summarized on a biweekly basis. Historical temperature and precipitation data was synthesized from daily summaries and reported on a biweekly basis as a line plot. Bounkiling and Ziguinchor are combined due to their proximity (< 100 km) and the availability of meteorological data only from Ziguinchor. Due to the unavailability of meteorological data directly from Tivaouane, data from the nearby (< 90 km) town of Diourbel is presented.

Plotting the collection dates of these putative arthropod-borne pathogen (arbopathogen) infections against historical weather data produces consistent trends (Figure 3). Typically, the mosquito- and louse-vectored pathogens were maximally detected during, or within the 3 months after the conclusion of, the annual monsoon. At the southern Bounkiling and Ziguinchor sites, these detections were highest after the 2020 monsoon, which brought more total rainfall than in 2021 or 2022. Unfortunately, sampling did not begin in Tivaouane until 2021, so no data is available for the 2020 monsoon, although it was not substantially wetter than those in 2021 or 2022. In contrast, 5/7 of the tick-borne *B. crocidurae* cases were detected during the dry season from January to August. The other pathogens (*e.g.*, bloodborne) did not follow a clear seasonal trend.

### Senegalese West Nile virus strains are related to those circulating in southern Europe and the United States

**FIGURE 4:**
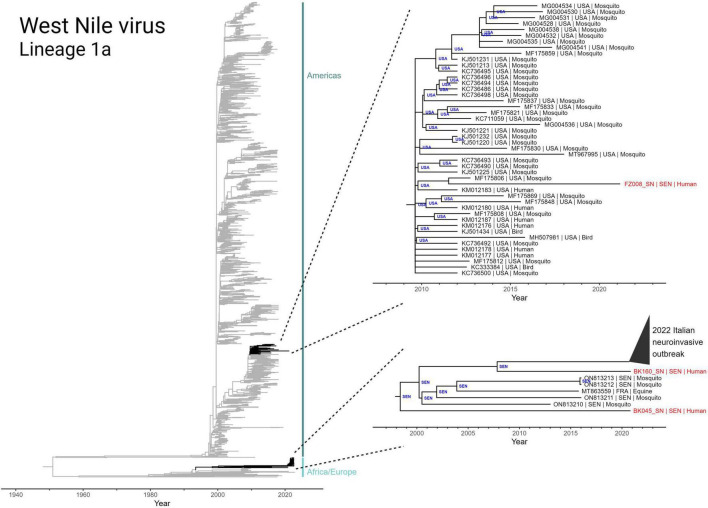
Discrete state phylogeographic analysis of lineage 1a of West Nile virus, incorporating the three Senegalese sequences (BK045, BK160, and FZ008) obtained in this study. A total of 2,156 full genome sequences were determined to conform to a molecular clock signal (GTR model; rate = 4.362 × 10^–4^ subs site^–1^ yr^–1^; *r*^2^ = 0.92) and are included in the tree. Highlighted clades (black) have tips annotated in the format “accession number | collection country | host” and nodes are annotated with inferred country of origin.

Due to the highly mobile nature of WNV, we utilized discrete phylogeographic analysis to reconstruct international transmission events in the evolutionary history of the three genomes collected in this study (Figure 4). We first inferred a maximum-likelihood molecular clock phylogeny of Lineage 1a of WNV (including the three new sequences) and rooted using the least-squares method. This phylogeny reveals a split in the 1950’s between an African/European-centric clade and a clade that eventually gave rise to the early 2000’s outbreak in the Americas.

The two WNV genomes collected from Bounkiling (BK045 and BK160) belong to the African/European-centric clade and are members of a nested clade that share a most recent common ancestor that emerged in 1998 (Figure 4, bottom right). Prior to 2020, members of this clade had only been detected in mosquitos from Senegal and a horse in France. BK045 and BK160, collected in 2021, were the earliest representatives in this clade to be detected in humans. Additionally, BK160 shares a most recent common ancestor in 2008 with a recently identified Italian cluster of WNV which was observed to cause a higher incidence of neuroinvasive symptoms in humans. Despite this connection, no neurological symptoms beyond headache were reported by the two affected patients. The discrete phylogeography infers a Senegalese origin of both the 2016 French equine case and the 2022 Italian human outbreak. Additionally, it indicates endemicity of this monophyletic group in Senegalese mosquitoes for well over 20 years. Additionally, analysis of branches basal to this monophyletic group (Nexus file available for view) reveals numerous geography-switching events between Senegal and Italy since at least 1980.

The WNV genome collected from Ziguinchor (FZ008), collected less than 100 km away from those in Bounkiling, occupies a different region of the Lineage 1a phylogeny, namely that of the Americas-centric clade. This sequence, collected in 2021, shares a most recent common ancestor (in 2011) with a strain found in an American mosquito. The genome of FZ008 represents the most recently collected member of a larger monophyletic group (Figure 4, upper right callout) that originated in late 2009 in the United States, responsible for infections in both birds and humans. Members of this monophyletic group have not been regularly detected since 2012–2015, indicating ongoing cryptic transmission in the years since.

### The combination of metagenomics and target enrichment allowed for the detection of co-infections and low concentration viruses

Coinfections were detected seven times in this study. Specimen BK208, collected from Bounkiling in November 2020 was infected with both *P. falciparum* and *R. felis*. This patient reported only mild fever, headache, and fatigue at the time of intake. Amongst the malaria-negative specimens, we detected an HIV/HBV dual infection (BK262), an HBV/*R. felis* dual infection (TV012), an HIV-1/*A. pullulans* dual infection (BK046), an *A. baumannii*/*A. pullulans* dual infection (BK099), a WNV/*A. baumanii* dual infection (BK160), and a *B. crocidurae*/*Loa loa* dual infection (BK324). The patient from which specimen BK324 was collected reported headaches and vomiting, consistent with *B. crocidurae* infection, but did not exhibit skin or ocular symptoms characteristic of *L. loa* infection during the study period.

The application of both target enrichment and metagenomic sequencing allowed for full genome recovery for many of the detected viral species (Table 2) and prevented missing some infections with presumably low viral load. For example, one WNV specimen (BK045) allowed for recovery of few NGS reads; target enrichment returned 91 reads, whereas metagenomics returned only 2, thus this infection could have easily been missed (or dismissed as misassignment or contamination) without target enrichment. The low read count, combined with this patient’s lack of fever, suggests that this is a case of resolving or resolved WNV infection. On the other hand, specimen BK160 allowed for the recovery of more than 300,000 WNV reads, with target enrichment providing a 45 × boost in RPM. In other examples like specimens BK120 or BK399, containing HBV and SAFV, respectively, target enrichment afforded 100% genome coverage and increased the coverage depth from < 10x to > 100x. Despite the sequencing depth available via these two methods, 178/228 specimens collected in this study (78%) remained without a pathogen identifiable in blood plasma. Unfortunately, the four comatose patients were also part of the 178 without an identified pathogen.

**TABLE 2:** NGS mapping statistics for pathogens identified in selected specimens.

Specimen ID	Pathogen	Closest reference	Identity (%)	CVRP-NGS				mNGS				Combined
				Coverage (%)	Coverage (avg. depth)	Mapped reads	RPM	Coverage (%)	Coverage (avg. depth)	Mapped reads	RPM	Mapping
BK046	HIV-1	L39106 (Nigeria)	91	99	98.2	9,087	169,066.8	98	16.9	2,192	86.1	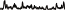
BK120	HBV	KX186854 (Guinea)	98	100	106.5	3,450	6,450.9	41	1.0	41	2.3	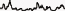
BK160	WNV	FJ483548 (Italy)	98	100	2,346.9	263,541	359,154.4	100	100.9	8,526	366.8	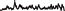
BK399	SAFV	MH933792 (Cameroon)	88	98	320.4	18,014	48,281.7	74	4.1	371	22.2	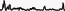
TV050	*B. crocidurae*	CP004267 (Mali)	98	1	0.1	503	2,545.7	95	18.2	220,025	12,374.6	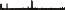
FZ006	HHV-8	OL829898 (Cameroon)	99	97	24.0	34,893	53,930.1	65	2.6	5,879	813.9	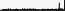

HIV-1: human immunodeficiency virus 1; HBV: hepatitis B virus; WNV: West Nile virus; SAFV: Saffold virus; *B. crocidurae*: *Borrelia crocidurae*; HHV-8: human gammaherpesvirus 8; RPM: specific pathogen reads per million total reads collected.

## Discussion

Globalization, travel, displacement of peoples, changes in the environment, and encroachment of humans into previously untouched habitats will increasingly contribute to infectious disease outbreaks in the future, testing the international capacity to effectively respond (Morens et al., 2020). The importance of the early detection and surveillance of disease events (Gostin and Katz, 2016) has prompted the formation of numerous scientific and public health networks (*e.g.*, (Mcsweegan et al., 2015; Martin and Fall, 2021) which seek to narrow the time required to achieve the early discovery of a pathogen, manufacture accurate diagnostics at scale, and trial treatment candidates. At the heart of these efforts lies case finding and scientific capacity building (Carroll et al., 2021), especially in developing countries where much of the emerging disease burden lies (Jones et al., 2008). Achieving these goals requires joint effort between academia, non-profits, the public health community, and industry partners.

AFI remains a major challenge for clinicians, both in identifying the correct etiology and providing successful treatment (Iroh Tam et al., 2016). Despite the wide introduction of malaria control measures, RDTs, and medication in sub-Saharan African countries, AFI is still common and can be caused by many non-malarial agents (Reyburn et al., 2004; Thiam et al., 2011; Wainaina et al., 2022). In this study, the three clinics in Senegal cumulatively tested over 7,000 patients for malaria using RDTs during the enrollment period and reported a 17% test positivity rate (Supplementary Table 1), comparable to the 18–25% positivity rate observed nationwide between 2016–2020 (U.S. President’s Malaria Initiative, 2022). These figures reinforce the notion that not all cases of AFI are malaria; supplemental testing strategies can help clinicians understand what other infections are in local circulation and serve as a warning system for new or re-emerging pathogens (Maze et al., 2018; Wainaina et al., 2022). Next-generation sequencing in a laboratory setting is an ideal platform for this research, as any pathogen can be detected in 1–3 days, with operating costs decreasing each year (Greninger, 2018; Chiu and Miller, 2019; Ko et al., 2022). With this goal, we assessed plasma collected from over 200 patients with negative malaria RDTs read at clinical intake (Figure 1) using metagenomic and viral target-enrichment methods. We identified sequencing reads corresponding to at least one human pathogen in 22% of these specimens (Figure 2).

In this cohort, we identified 8 species of viruses, 4 of bacteria, 1 of fungus, and 2 of parasites. Those transmitted by mosquitos were detected during or just after the annual monsoon (Figure 3), consistent with observations across multiple other studies (see a review in Agboli et al., 2021). The viral pathogens were diverse, spanning multiple families including flaviviruses, coronaviruses, herpesviruses, picornaviruses, and others. Based on the numerous regional reports available using targeted diagnostics like RT-qPCR (see a review in Wainaina et al., 2022), arboviruses such as Dengue, Chikungunya, Yellow Fever, or Rift Valley Fever (all readily detectible in plasma) were expected to be present, but were missing from our cohort. This observation (or lack thereof) was likely due to many arboviral infections recurring in cycles separated on the scale of months to years; our sampling simply did not overlap in time or space with an outbreak. Nevertheless, a benefit of metagenomic NGS is its ability to subvert availability bias and detect any pathogen present in a cohort, regardless of whether it is pre-conceived to be rare or common.

Our study supports the increasing recognition of TBRF as a significant cause of AFI in West Africa. While Senegal reports the most cases in the literature, data exist of the presence of *B. crocidurae* in other countries in the region such as Mali, Mauritania, Morocco, and Algeria (Trape et al., 2013). Our 3% detection rate of *B. crocidurae* in AFI cases (7/228 specimens) was higher than a previous estimate of < 0.5% (Brahim et al., 2005). Additionally, with the discovery of three patients from the southern Bounkiling clinic (13.04 N latitude) with evidence of *B. crocidurae* infection, the geographic range of TBRF in Senegal has been pushed further south than has ever been observed before (in humans or animals) (Trape et al., 2013; Gras et al., 2019). Additionally, its clinical presentation within our cohort was nearly identical to that of malaria (Table 1), which, combined with its low culturability and low detection sensitivity by microscopy, could confound proper diagnosis and treatment. The time of year in which TBRF was identified differed between the sites in the north (Tivaouane) and the south (Bounkiling); in the north, the three cases were found between May-July, whereas the four in the south were identified between October-February. Interestingly, one of the affected patients in Bounkiling (BK324) was likely co-infected with the filarial parasite *L. loa*. This parasite is considered endemic to central Africa (Kelly-Hope et al., 2018), though there is documentation of travelers from that region being diagnosed with loiasis in Senegal [for example, Diagne et al. (2018)]. Despite the lack of specific symptoms (*i.e.*, no recorded skin lesions or ocular disturbances), the NGS reads recovered for *L. loa* in this specimen were numerous enough (299 total reads, or 17.02 specific reads per million sequenced) to lend confidence to this finding.

The only mosquito-transmitted virus detected in this study was WNV. Our phylogenetic analysis of the three detected strains demonstrates that WNV circulation within Senegal is affected by transmission chains involving both European and North American nations (Figure 4). While the importance of North American-to-African transmission of WNV is unclear and requires further study, the repeated observations of transmission between Europe and Africa can be explained by yearly bird migrations on the Mediterranean-Sahel flyway (Hahn et al., 2009; Najdenski et al., 2018; García-Carrasco et al., 2022). Our maximum-likelihood molecular clock phylogeny and accompanying discrete phylogeographic analysis (Figure 4, bottom right) also revealed the relatedness of the WNV strains found in Bounkiling with at least 39 strains recently causing a neuroinvasive outbreak in northern Italy (Barzon et al., 2022a,b). Disconcertingly, the tree also exposes the need for additional genomic surveillance: the clade containing the Italian and Bounkiling strains (particularly BK160) shared a common ancestor more than 15 years ago, without any other genomic sequences collected during the interim. Without ongoing collection of genomic data, increases in genetic diversity or effective reproduction rate (which in turn increase pathogen fitness) cannot be quantified, preventing health authorities from anticipating or reacting to potential outbreaks.

A major confounding factor in the diagnosis of AFI is the occasional occurrence of coinfection. In our study, we identified seven such cases. In particular, the *P. falciparum*/*R. felis* coinfection is concerning because it would have been missed by our collection program had we not retained some malaria positives; thus, focusing on malaria negative specimens alone will not provide a full picture of the extent of non-malaria AFI etiologies. On the other hand, we also suggest providing continuing training for clinicians on correct RDT usage to avoid misdiagnosis, especially when reflex microscopy is unavailable or when RDTs with low sensitivity are used. The detection of a range of coinfections across multiple pathogen types (i.e., viral, bacterial, parasitic, fungal) was only possible due to our use of unbiased NGS. Though we looked for divergent pathogens (particularly viruses) and did not find any, our unbiased NGS methods and prediction algorithms are equipped to detect them (Berg et al., 2015, 2021; Orf et al., 2023a,b).

A limitation of this study was inconsistent recruitment of patients over the study period, along with a relative lack of enrolled children. We seek to remedy this situation in the future through better communication with clinic personnel and potential enrollees (or their parents/guardians). We also plan to extend collections to new sites to represent most ecoregions of the country. Another limitation of this study was its reliance on the collection of plasma alone; this reduces the ability to detect AFI-causing enteric or respiratory pathogens, or bacteria (opportunistic or pathogenic) generally. This inevitably results in an underrepresentation of bacterial and parasitic causes of AFI. Despite the burden that non-respiratory pathogens put on healthcare systems, the recent COVID-19 pandemic reinforced that human-to-human respiratory transmission still poses the greatest risk for the rapid dissemination of a novel pathogen. In the future, we plan to expand the number of matrixes collected from patients enrolled in our surveys and initiate follow-up collections to increase the chance of detecting an infection at a high titer. Encouragingly, since viral target enrichment enables the recovery of sequence at low concentration (Table 2), this increases the window during the viral infection cycle that a successful detection can be achieved in a patient. The strengths of viral target enrichment also allowed us to collect full genomes for, and thus highlight, lesser-appreciated and under-studied viral infections such as Saffold virus and HHV-8. In the case of the latter, the prevalence of AFI caused by herpesviruses will be interesting to monitor going forward due to the observation that COVID-19 can reactivate latent infection (Shafiee et al., 2023).

The efforts described in this study embody the objectives of the Abbott Pandemic Defense Coalition (Averhoff et al., 2022). Senegal represents an ideal setting for conducting case finding and pathogen identification for non-malarial febrile illnesses. Collaboration between established scientific programs in developed countries and those in developing countries will be necessary to identify new or re-emerging pathogens and respond appropriately. We believe that implementing metagenomic and target-enriched sequencing technology locally where the highest burden of disease exists will decrease turnaround time and encourage stronger relationships between local scientists, clinicians, and health authorities.

## Data availability statement

The data presented in the study (viral genomes with greater than 50% coverage) are deposited in the NCBI GenBank repository, accession numbers PP445045-PP445060.

## Ethics statement

The studies involving humans were approved by the National Ethics Committee for Health Research (Comité National d’Ethique pour la Recherche en Santé; CNERS) of the Senegalese Ministry of Health, under approval number 000129/MSAS/CNERS. The studies were conducted in accordance with the local legislation and institutional requirements. Written informed consent for participation in this study was provided by the participants’ legal guardians/next of kin.

## Author contributions

GO: Data curation, Formal analysis, Investigation, Methodology, Resources, Software, Visualization, Writing−original draft, Writing−review and editing. AA: Conceptualization, Data curation, Investigation, Methodology, Resources, Validation, Writing−original draft, Writing−review and editing. MM: Formal analysis, Investigation, Writing−review and editing. CD: Investigation, Resources, Writing−review and editing. AM: Resources, Writing−review and editing. AP: Investigation, Resources, Writing−review and editing. NM: Resources, Writing−review and editing. AD-d: Resources, Writing−review and editing. FA: Conceptualization, Writing−review and editing. MB: Conceptualization, Methodology, Project administration, Resources, Software, Supervision, Writing−review and editing. GC: Conceptualization, Funding acquisition, Project administration, Resources, Supervision, Writing−review and editing. SM: Conceptualization, Funding acquisition, Project administration, Supervision, Writing−review and editing.

## Footnotes

1
https://www.ncei.noaa.gov/cdo-web/datasets/GHCND/locations/FIPS:SG/detail
