# Supplementary material for: Next-generation sequencing survey of acute febrile illness in Senegal (2020–2022)
Source: Front Microbiol. 2024 Apr 9;15:1362714. doi: 10.3389/fmicb.2024.1362714 (PMC11037400; doi:10.3389/fmicb.2024.1362714)
Supplement: Supplementary file 1 [file Data_Sheet_1.PDF]

Supplementary Information for: Next-generation sequencing survey of acute febrile illness in Senegal (2020-2022)

Gregory S. Orf[1,2,†,*], Ambroise D. Ahouidi[2,3,†], Maximillian Mata[1,2], Cyrille Diedhiou[2,3], Aminata Mboup[3], Abdou Padane[2,3], Noel Magloire Manga[4], Agbogbenkou Tevi Dela-del Lawson[5], Francisco Averhoff[1,2], Michael G. Berg[1,2], Gavin A. Cloherty[1,2], and Souleymane Mboup[2,3]

[1] Abbott Diagnostics Division, Abbott Laboratories, Abbott Park, IL, USA
[2] Abbott Pandemic Defense Coalition, Abbott Park, IL, USA
[3] Institut de Recherche en Santé, de Surveillance Epidémiologique et de Formation, Diamniadio, Dakar, Senegal
[4] Unit of Infectious and Tropical Diseases, Université Assane Seck, Hôpital de la Paix, Ziguinchor, Senegal
[5] Unit of Infectious and Tropical Diseases, Hôpital Mame Abdou Aziz Sy Dabakh, Tivaouane, Senegal
[†] These authors share first authorship
[*] To whom correspondence should be addressed: gregory.orf@abbott.com

Supplementary Results

We evaluated the necessary Sample Size using the standard Sample Size formula for infinite populations: $n = \frac{z^2 \times p(1-p)}{\varepsilon^2}$, where n is the Sample Size, z is the z-score, ε is the margin of error, and p is the population proportion. In all, 7,137 patients with AFI were received at the clinics and 83% of these tested negative and were eligible for screening by NGS (**Table S1** below). With these values, we calculated that 217 specimens would need to be collected to have a confidence level of 95% (z-score of 1.96) and margin of error of 5% (0.05) that the results generated from the collected specimens could be generalized to the populations seen at the clinics. Thus, with 228 specimens collected in this study, we exceed this number.

Table S1: Accounting of malaria testing and study enrollees during the study period. Collections occurred from Oct 2020-Jul 2022 in Bounkiling, from Nov 2020-Apr 2021 in Ziguinchor, and from Aug 2021-Jul 2022 in Tivaouane.

Clinic	Month	Malaria RDTs administered	Malaria RDT positives	Study enrollees
Bounkiling	Oct 2020	708	254	46
	Nov 2020	425	297	15
	Dec 2020	110	23	0
	Jan 2021	89	11	14
	Feb 2021	112	2	2
	Mar 2021	88	0	0
	Apr 2021	77	0	4
	May 2021	60	0	17
	Jun 2021	60	0	9
	Jul 2021	98	6	6
	Aug 2021	172	16	3
	Sep 2021	117	29	0
	Oct 2021	349	160	3
	Nov 2021	264	77	0
	Dec 2021	397	151	2
	Jan 2022	93	14	24
	Feb 2022	70	6	10
	Mar 2022	49	3	0
	Apr 2022	34	2	0
	May 2022	68	0	0
	Jun 2022	60	0	0
	Jul 2022	97	17	15
Ziguinchor	Nov 2020	403	55	1
	Dec 2020	73	24	2
	Jan 2021	140	6	3
	Feb 2021	131	6	6
	Mar 2021	139	7	1
	Apr 2021	117	0	1
Tivaouane	Aug 2021	168	2	1
	Sep 2021	232	5	2
	Oct 2021	457	19	11
	Nov 2021	311	5	3
	Dec 2021	188	0	7
	Jan 2022	226	1	4
	Feb 2022	182	2	3
	Mar 2022	194	1	0
	Apr 2022	123	1	0
	May 2022	138	1	9
	Jun 2022	157	4	8
	Jul 2022	161	5	3

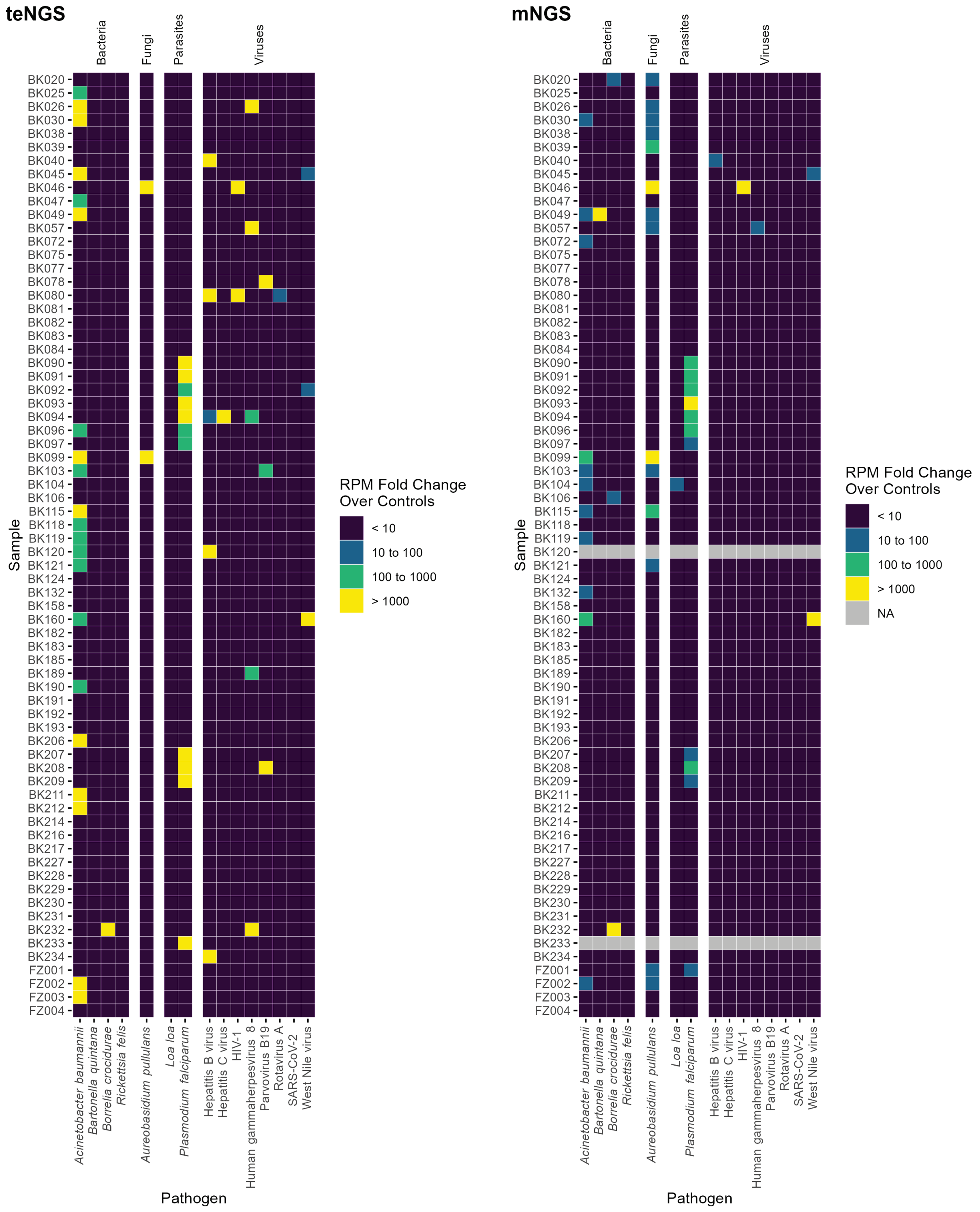

Figure S1: NGS-based identification of pathogen nucleic acids in specimens collected in 2020. Shown is a heatmap representation of pathogen NGS hits, normalized in reads per million (RPM) against reads found in the no-template control (NTC), which is called "RPM ratio" (RPM-r) or "RPM fold change above controls". See main text for information regarding the calculation of the RPM-r metric. Results from both teNGS and mNGS are shown.

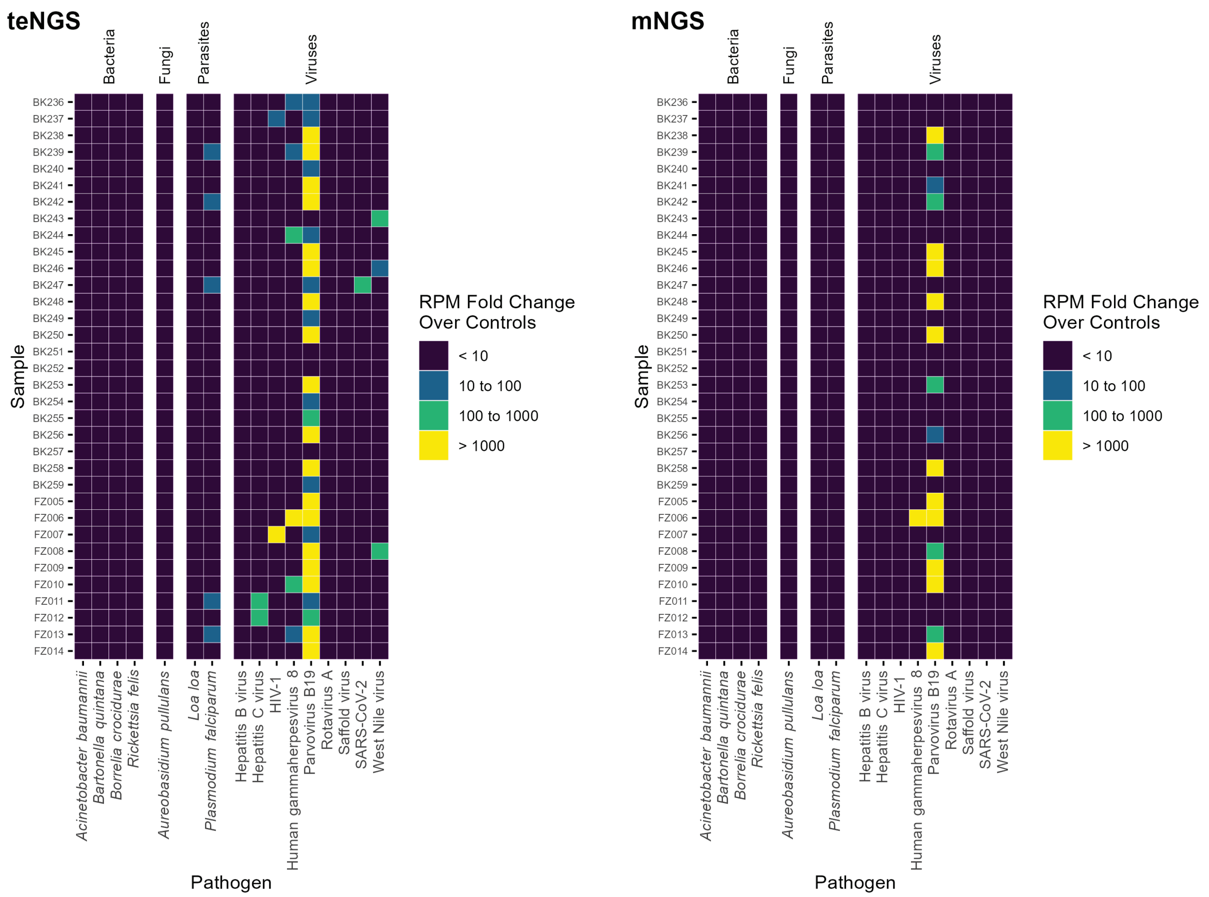

Figure S2: NGS-based identification of pathogen nucleic acids in specimens collected in 2021. The same analysis and heatmap representation is shown as in Figure S1.

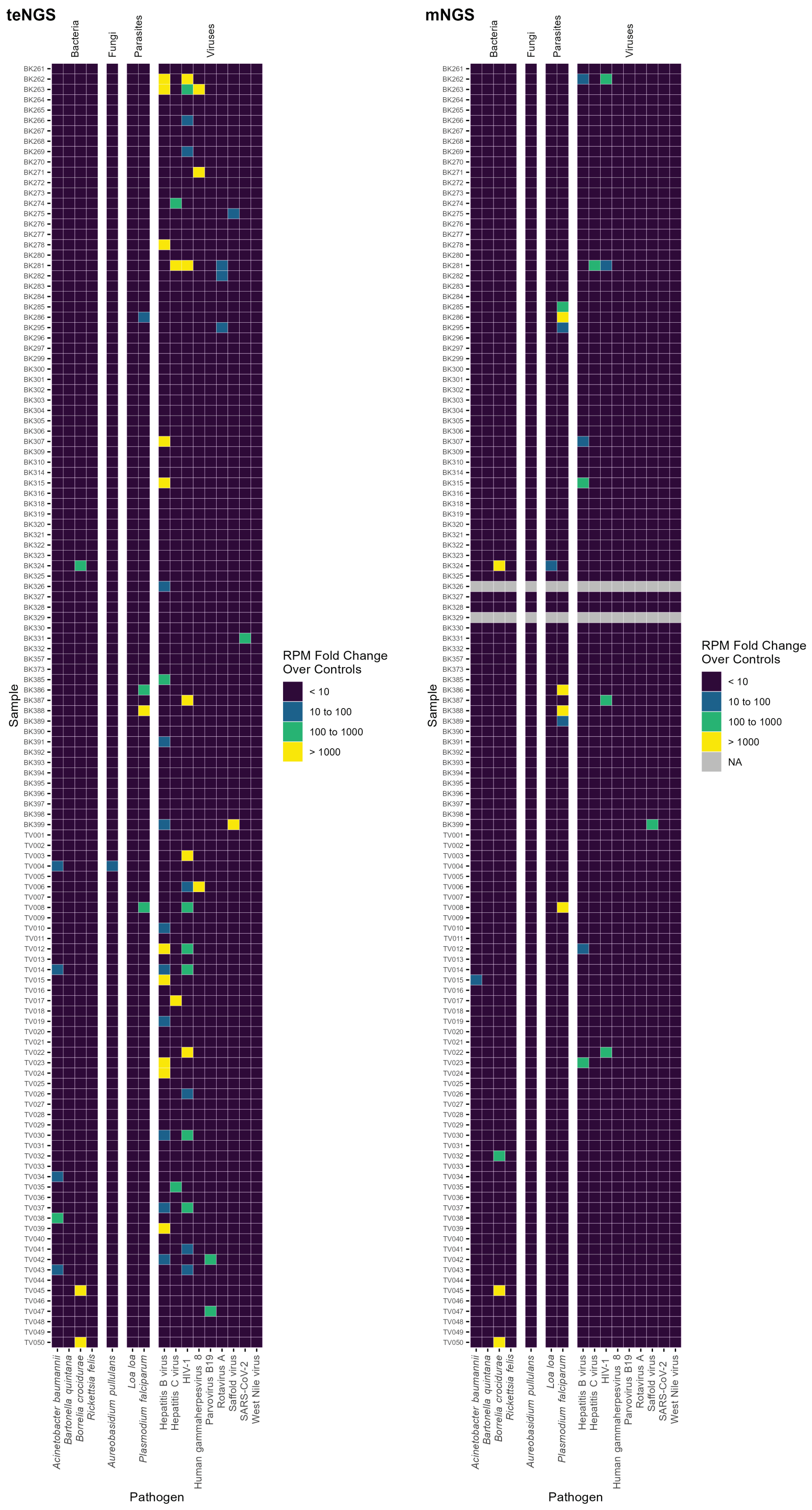

Figure S3: NGS-based identification of pathogen nucleic acids in specimens collected in 2022. The same analysis and heatmap representation is shown as in Figure S1.
